# High-resolution hydrometeorological data from a network of headwater catchments in the tropical Andes

**DOI:** 10.1038/sdata.2018.80

**Published:** 2018-07-03

**Authors:** Boris F. Ochoa-Tocachi, Wouter Buytaert, Javier Antiporta, Luis Acosta, Juan D. Bardales, Rolando Célleri, Patricio Crespo, Paola Fuentes, Junior Gil-Ríos, Mario Guallpa, Carlos Llerena, Dimas Olaya, Pablo Pardo, Gerver Rojas, Marcos Villacís, Mauricio Villazón, Paúl Viñas, Bert De Bièvre

**Affiliations:** 1 Imperial College London, Department of Civil and Environmental Engineering & Grantham Institute – Climate Change and the Environment, London SW7 2AZ, UK.; 2 Regional Initiative for Hydrological Monitoring of Andean Ecosystems (iMHEA), Lima 15024, Peru.; 3 Consorcio para el Desarrollo Sostenible de la Ecorregión Andina (CONDESAN), Área de Cuencas Andinas, Lima 15024, Peru.; 4 Superintendencia Nacional de Servicios de Saneamiento (SUNASS), Gerencia de Regulación Tarifaria, Lima 15073, Peru.; 5 Universidad de Cuenca, Departamento de Recursos Hídricos y Ciencias Ambientales (iDRHiCA), Facultad de Ciencias Agropecuarias, Facultad de Ingeniería, Cuenca 010203, Ecuador.; 6 Fideicomiso Mercantil Fondo Ambiental para la Protección de Cuencas y Agua (FONAG), Secretaría Técnica & Programa de Recuperación de la Cobertura Vegetal, Quito 170137, Ecuador.; 7 Empresa Pública Municipal de Telecomunicaciones, Agua Potable, Alcantarillado y Saneamiento de Cuenca (ETAPA EP), Subgerencia de Gestión Ambiental, Cuenca 010101, Ecuador.; 8 Universidad Nacional Agraria La Molina (UNALM), Facultad de Ciencias Forestales, Lima 12056, Peru.; 9 Naturaleza y Cultura Internacional (NCI), Piura 20009, Peru.; 10 Universidad Mayor de San Simón, Laboratorio de Hidráulica (LHUMSS) & Facultad de Ciencias y Tecnología, Cochabamba 6760, Bolivia.; 11 Asociación Peruana para la Conservación de la Naturaleza (APECO), Chachapoyas 01001, Peru.; 12 Escuela Politécnica Nacional (EPN), Departamento de Ingeniería Civil y Ambiental, Quito 170525, Ecuador.

**Keywords:** Hydrology, Environmental chemistry, Hydrology, Water resources, Environmental chemistry

## Abstract

This article presents a hydrometeorological dataset from a network of paired instrumented catchments, obtained by participatory monitoring through a partnership of academic and non-governmental institutions. The network consists of 28 headwater catchments (<20 km^2^) covering three major biomes in 9 locations of the tropical Andes. The data consist of precipitation event records at 0.254 mm resolution or finer, water level and streamflow time series at 5 min intervals, data aggregations at hourly and daily scale, a set of hydrological indices derived from the daily time series, and catchment physiographic descriptors. The catchment network is designed to characterise the impacts of land-use and watershed interventions on the catchment hydrological response, with each catchment representing a typical land use and land cover practice within its location. As such, it aims to support evidence-based decision making on land management, in particular evaluating the effectiveness of catchment interventions, for which hydrometeorological data scarcity is a major bottleneck. The data will also be useful for broader research on Andean ecosystems, and their hydrology and meteorology.

## Background & Summary

Tropical mountain environments host some of the most complex, dynamic, and diverse ecosystems^1,2^, but are under severe threats, ranging from local deforestation and soil degradation^3–5^ to global climate change^6–8^. Despite their importance and vulnerability, there are very few observational data to quantify the impacts of these changes^9^. More extensive observations of climatic conditions at high elevations are urgently needed but monitoring in these areas is expensive and logistically challenging^10,11^. Despite many recent monitoring efforts to fill data gaps in high-altitude environments^12^, many mountain areas still lack a strong evidence base to guide decision-making on catchment interventions, such as re- or afforestation, and different conservation and climate adaptation measures. Driven by global agenda that promotes a better use of ecosystem services and natural capital, such interventions are happening at an accelerating pace, yet their hydrological benefits are insufficiently monitored and often based on anecdotal evidence. This is particularly the case for the tropical Andes, where the complex climatic and hydrological characteristics combined with a very dynamic anthropogenic disturbance put severe pressure on water resources^13^.

This combination of high vulnerability, accelerated change, and data scarcity makes it paramount to explore alternative methods of data collection. Among those, the use of participatory and grassroots approaches of information generation is gaining traction^14,15^. Participatory data collection has the potential to enhance public involvement and to fill long-standing data gaps^16–18^. It also has the potential to complement another major advance in hydrometeorological monitoring, remotely sensed data^19–22^, which are still limited by low spatial and temporal resolutions —usually kilometric areal averages and multi-daily— that impede hydrological assessment of small headwater catchments. Satellite precipitation products, for instance, are available at intra-daily timescales, but they involve large uncertainties and inaccuracies at the point-area difference with respect to surface level rain gauges^23,24^ that difficult their direct assimilation.

**Figure 1:**
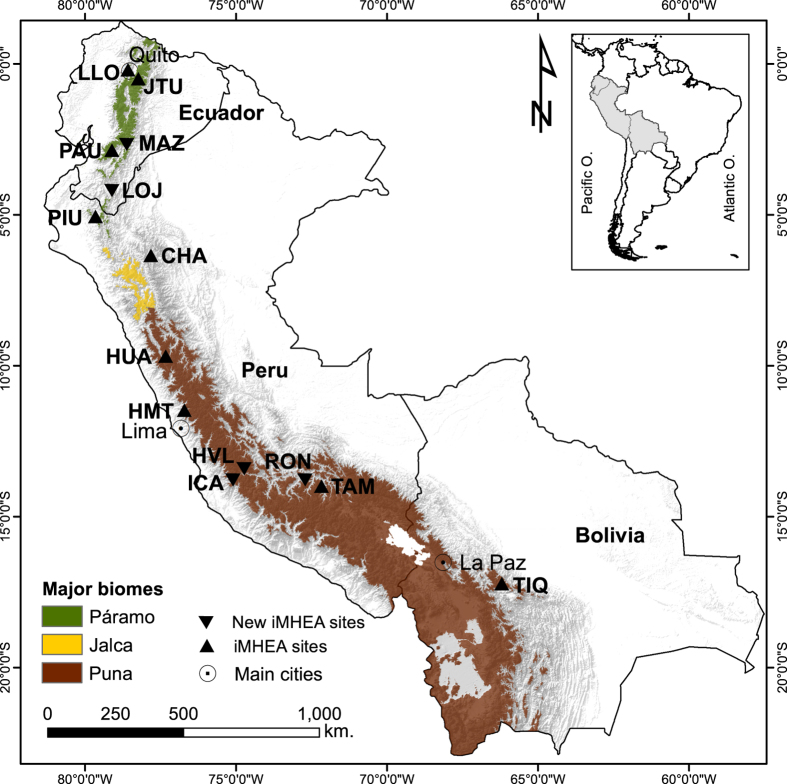
Map of the Regional Initiative for Hydrological Monitoring of Andean Ecosystems (iMHEA). The monitoring sites cover the major high Andean biomes (Páramo, Jalca, and Puna) in 3 countries (Ecuador, Peru, and Bolivia). The complete iMHEA network consists of 16 sites, including one in the Venezuelan Andes. In this article, we present data from the 9 sites identified in the map by up-pointing triangles.

As a response to such a monitoring gap, a partnership of academic and non-governmental institutions set up an initiative for participatory hydrological monitoring in the Andes^11,25^. The network known as *Regional Initiative for Hydrological Monitoring of Andean Ecosystems* (iMHEA) aims to produce information about the impacts of land-use and watershed interventions on hydrological ecosystem services, and to guide processes of decision making on catchment management. Similar bottom-up initiatives have emerged worldwide creating networks of well-monitored experimental sites^26^, for example, the network of terrestrial environmental observatories in Germany (TERENO)^27^. The iMHEA partnership, established in 2009, uses a hydrological design based on a ‘trading-space-for-time’ approach^28–33^. This concept relies on strengthening the statistical significance of a watershed intervention, such as a land-use signal, by monitoring several paired catchments in a regional setting^5^. The setup of the monitoring sites also allows for robust regionalisation results by covering different ecosystems with diverse physiographic characteristics and contrasting land uses and degrees of conservation/alteration^34^. This paper presents data from 28 catchments from the iMHEA network, representing 16 local stakeholders in 9 sites located in Ecuador, Peru, and Bolivia (Fig. 1). The presented data are composed of precipitation event records at 0.2 mm resolution, water level and streamflow time series at 5 min intervals, data aggregated at hourly and daily scale, as well as a set of hydrological indices derived from the daily time series, and catchment physiographic descriptors and geographic information.

The NGO CONDESAN and Imperial College London co-designed and implemented several iMHEA sites with local stakeholders as part of the Mountain-EVO research project (UK NERC grant NE-K010239-1) “Adaptive governance of mountain ecosystem services for poverty alleviation enabled by environmental virtual observatories” funded under the Ecosystem Services for Poverty Alleviation programme (www.espa.ac.uk) from 2013 to 2017. The project analysed how monitoring and knowledge generation of ecosystem services in mountain regions can be improved and used to support a process of adaptive, polycentric governance focused on poverty alleviation^16,17^. The data reported here fill long-standing hydrological information gaps allowing for evidence-based, robust predictions and cost-benefit comparisons about the effectiveness of watershed interventions. Such an approach has the potential to alleviate data scarcity in other remote mountain environments and provide relevant input for multi-scale decision making —from community level to national governance entities— ultimately improving the management of water resources and hydrological ecosystem services.

## Methods

### Hydrometeorological monitoring design

**Figure 2:**
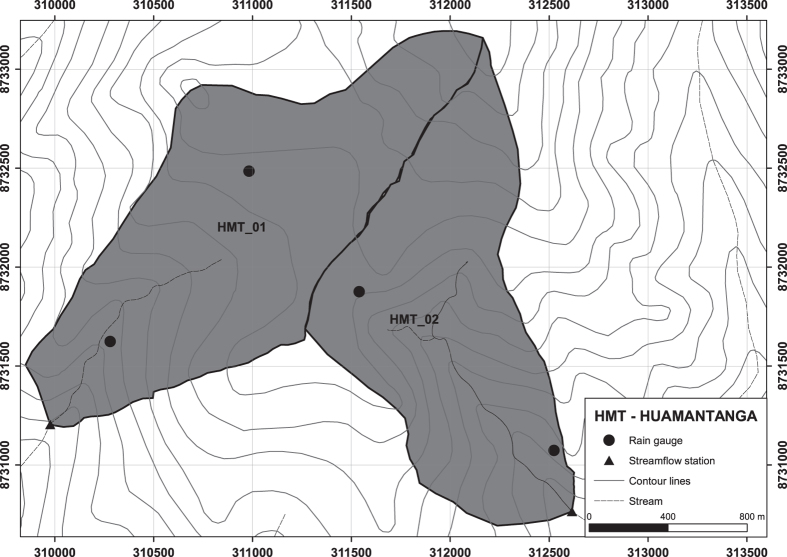
Example of paired catchments monitored at the iMHEA site HMT. Catchments are selected in such a way that they are as physically similar possible. The monitoring system is designed to capture small scale variability in hydrometeorological characteristics. Maps of all catchments are available in the geographic dataset (Data Citation 1). For details of catchment characteristics see Table 1.

A monitoring protocol was designed to optimise the value of the resulting dataset to evaluate the impact of catchment interventions which is available in Ochoa-Tocachi *et al.* (2017, ref. 25). The protocol is based on a pairwise catchment comparison (Fig. 2) to separate the effects of changes in land use or watershed interventions from those of natural variability in climate and physiography^35–37^.

An idealised paired catchment experiment consists of two catchments that are collocated or in close proximity to each other, and whose physical and climatic characteristics are as similar as possible^38^. These catchments are monitored for a sufficient time to generate a statistical baseline for comparison. Subsequently, one of the catchments is subject to an intervention while the second remains in the original (reference) state. This makes it possible to evaluate the hydrological impact of the intervention, even if boundary conditions –such as climate– change over time. Although the use of a statistical baseline provides the most robust design, this is generally only achievable under strict experimental control of both catchments, which is often not practical. Therefore, a more straightforward ‘control-intervention’ setup (i.e. without baseline) is more commonly implemented instead, and has proven useful in published case studies^5,38,39^.

A major advantage of the ‘control-intervention’ setup is that impacts are evaluated by means of a spatial comparison rather than of changes over time, which reduces the length of the monitoring times, but this comes at the expense of a reduced attribution power. Therefore, a careful analysis and interpretation is needed to avoid any incorrect attribution of hydrological differences that may not be caused by the controlled factor but by other variables^34,40^. On the long-term, nonetheless, as the length of the monitoring time series increases^26^, individual catchments can be evaluated for impacts of climatic changes or vegetation physiology, especially in the evaluation of restoration strategies. The long-term sustainability of the monitoring using the paired catchment design can therefore reveal meaningful insights about hydrological non-stationarity. Such sustainability can be achieved by a robust institutional agreement as highlighted below in this article.

In each catchment, we monitored rainfall and streamflow, and measured or calculated several catchment physiographic characteristics (Data Citation 1). In extension, we applied a catchment water balance equation on the dataset of precipitation and discharge to estimate the combined losses, which account for an estimation of total water consumed by vegetation, evaporated from the surface, infiltrated to deeper soil strata, and stored in the soils. On the long-term, the water balance becomes a good approximation of catchment evapotranspiration. However, deep permeable soils and aquifers may be present in some regions^3,41,42^ which may challenge the assumption of a closed water balance. In such cases, a careful identification of the catchments has been performed to minimise errors and potential issues in the water balance simplification, or to monitor and consider those elements in the analyses.

### Catchment physiography

Surveys of physical features were performed to select catchments and to document the properties that can influence their hydrological response. These properties were derived from geographic and cartographic data^34^, and divided in categories of shape (area, perimeter, compactness, shape factors, circularity, elongation, equivalent rectangle dimensions), drainage (longest drainage path, longest line parallel to the main stream, drainage density, current frequency, concentration time), elevation (maximum, minimum, mean, range, weighted average, percentiles), topography (hypsometric curve, catchment mean slope, stream mean slope, relief ratio, slope variability, topographic index), subsurface (soil types, soil depths, mean hydraulic conductivity, geology), land cover percentages, and land use types.

**Figure 3:**
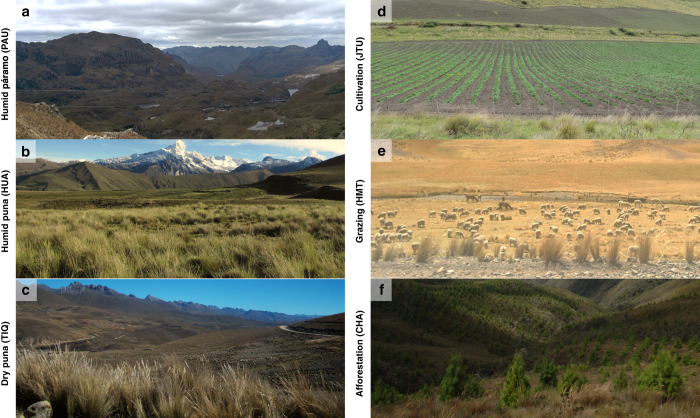
Representative landscapes of the monitored catchments. The iMHEA network covers major high Andean biomes, including (**a**) Humid páramo in southern Ecuador (e.g., PAU), (**b**) Humid puna in central Peru (e.g., HUA), and (**c**) Dry puna in central Bolivia (e.g., TIQ). Land use types include common human activities such as (**d**) cultivation of potato and tubers (e.g., JTU), (**e**) livestock grazing (e.g., HMT), and (**f**) afforestation with exotic tree species (e.g., CHA). For a reference of site codes and locations, see Fig. 1 and Table 1.

A summary of the main catchment characteristics is shown in Table 1. The monitoring sites cover three of the most extensive upper Andean biomes^5^, i.e. páramo, jalca, and puna (Figs 3a–c), with some catchments also covered by high Andean forests. Catchments are small, ranging from 0.5 km^2^ to 20 km^2^, and located between 2000 m and 5000 m altitude. They feature a typical mountainous topography, with steep and uneven slopes, and oval shapes tending to circular or stretched. Sites are rural and the main land use types are conservation, grazing, afforestation, and cultivation (Figs 3d–f). Vegetation cover is generally natural and mostly consists of tussock and short grasses, shrubs patches with eventual occurrences of native and exotic forest, and small wetlands in the flat valley bottoms. Catchments classified as forested have at least 80% of their area under forest cover. No water abstractions or alterations are present upstream of the streamflow monitoring stations.

### Precipitation

Rainfall is the main component of precipitation in Andean catchments^43^. Precipitation was measured using electronic tipping bucket rain gauges (Onset HOBO Data Logging Rain Gauge, Davis Instruments Rain Collector II, or Texas Electronics Collector Rain Gauge), installed at a height above the ground of 1.50 m. Their resolution is of at least 0.254 mm (0.1 in), but typically 0.2 mm, and exceptionally 0.1 mm. At least two rain gauges were located to improve capturing the catchment area and its elevation range, which is known to generate high spatial variability^44,45^. Furthermore, locations were selected for regional representativeness and to minimise measurement errors such as those caused by wind effects^46–48^. Correlation analysis and double mass plots between the rain gauges were used to detect and correct errors and to fill data gaps. Automatic precipitation records were validated using manual rain gauges. In areas with potential snowfall occurrence, the equipment is above the maximum expected snow height accumulation in the ground, and an antifreeze substance has been recommended to melt ice and snow^25^. However, snowfall is rare within the monitored elevation range in the tropical Andes and has not been reported by local monitoring operators.

**Figure 4:**
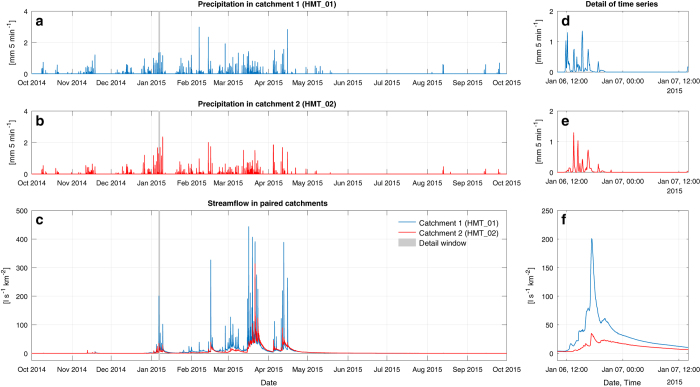
Representative sample of the generated time series. Precipitation (**a**,**b**) and streamflow (**c**) data from the paired catchments at the iMHEA site HMT (see Fig. 2). The variables are monitored at high resolution, which allows for a very detailed characterisation of hydrometeorological regimes (**d**–**f**).

Tip time stamps were transformed to rainfall volumes depending on the rain gauge resolution. A composite cubic spline interpolation^49,50^ was applied to the cumulative rainfall curve to generate rainfall intensities at 1 min resolution. This procedure generates better results than simple tip counting or linear interpolation^51^ (Fig. 4). Rainfall data were then aggregated at time intervals matching those of the corresponding discharge in the catchment, and also at 1 hr and 1 day resolutions. From these data, we derived summarising indices including average annual precipitation, wetness index, days with zero rainfall, precipitation of the driest month, coefficient of variation in daily precipitation, and seasonality index^52^. For the calculation of median and maximum rainfall intensities, we used a 5 min scale moving window for storm durations between 5 min and 2 days. As most of the catchments lack a full meteorological station, the wetness index (ratio between annual precipitation and evapotranspiration) was calculated using WorldClim temperature data^53^ and the Hargreaves formula^54,55^.

### Streamflow

Discharge was obtained by measuring water level using automatic pressure transducers (Onset HOBO U20 Water Level Loggers, Schlumberger Water Services Diver and Baro-Diver, Solinst Levelogger 3001, Global Water WL16, or Instrumentation Northwest AquiStar PT2X Smart Sensor). Water level was recorded at a regular interval of at least 15 min and generally 5 min, although some catchment data are limited to 30 min intervals. The measurement resolution ranges between 0.1 to 0.5 mm. The high temporal resolution of the recordings allows capturing the rapid hydrological response of small catchments after precipitation events, which can reach peak flows in only a few minutes (Fig. 4).

Compound sharp-crested weirs were used to rely on an established stage-discharge relation^47^. A compound weir combines a V-shaped section for accurate monitoring of minimum flows, and a triangular–rectangular section to contain peak flows. Manual flow velocity measurements were used to cross-check automatic water level records. The Kindsvater–Shen relation^56^ was used to transform water level to discharge, and then revised with flow observations. Discharge data were normalised by catchment area (units of mm or l s^–1^ km^–2^) to enable comparison between catchments and water balance calculations with rainfall. These data were kept at the maximum temporal resolution and averaged at 1 hr and 1 day resolutions, from which a set of hydrological indices was computed.

### Derived hydrological indices

The hydrological literature offers a large number of indices (also known as streamflow signatures) that characterise different features of the hydrological regime^57–59^. Indices are useful to represent several conservation/alteration conditions in catchments by means of streamflow information. Streamflow data are typically analysed in terms of five major response characteristics: (i) Magnitude (flow at any given time interval and location); (ii) Frequency (how often a flow above a threshold recurs over time); (iii) Duration (period of time over which a specific flow condition exists); (iv) Timing/Predictability (regularity with which a flow of a predefined magnitude occurs); and, (v) Rate of change/Flashiness (how quickly the flow magnitude changes). These characteristics can be subdivided further in low, average, and high flows.

We selected hydrological indices that are independent^37,60^, unambiguous^59,61^, and respond to the contextualised practical issue^34,62^, i.e., those that show evidence of change under watershed interventions, land use changes, or green infrastructure implementation. iMHEA partners are particularly interested in characterising water yield (flow statistics such as minimum, maximum, median, annual mean, long-term mean, driest month flow, and flow percentiles), hydrological regulation (baseflow index, recession constant, discharge range, slope of the flow duration curve, hydrological regulation index, Richards-Baker flashiness index^63^), and water balance (dry month discharge ratio, dry month discharge range, average annual discharge, runoff ratio). This list has been further expanded to include selected indices^34,59^ of magnitude (skewness in daily flows, coefficients of variation, discharge ranges), high and low flow pulse analysis (magnitude, volume, frequency, and duration), timing (flood occurrence or absence, Julian day of extremes), and flashiness (flow reversals, logarithm of increasing and decreasing flow differences, ratio of days with increasing flow). We developed scripts to derive the several hydrological indices from normalised daily discharge data^34^.

One particular interest in the monitoring of small and seasonal catchments is the characterisation of low flow conditions. The Base Flow Index (BFI), calculated as the ratio between baseflow to total flow, is interpreted as the proportion of river discharge that originates from internal catchment stores^64^. We followed two different procedures to derive baseflow time series and the BFI. The first method follows the UK Flood Estimation Handbook^64^ dividing the mean daily flow data into non-overlapping blocks of five days. The method calculates the minima of these consecutive periods and subsequently searches for turning points in this sequence. Daily values of baseflow are calculated by linear interpolation between these turning points and constrained by the original hydrograph when the separated hydrograph exceeds the observed. Baseflow time series are included in the daily flow dataset (Data Citation 1). The second method follows the Boughton two-parameter algorithm^65^ with a filter parameter of 0.85 that is fitted subjectively. The two-parameter algorithm provides more consistent results than either a one-parameter algorithm based on the recession constant or than the IHACRES three-parameter algorithm^65^. We identified baseflow recessions as sections of the hydrograph of at least a 7 day duration that were apparently linear (R^2^>0.8) on a logarithmic scale for flows, from which the hydrograph recession constant was calculated. The presented hydrological indices include BFI and recession constants obtained by the two methods.

### Institutional agreement for participatory monitoring

The monitoring sites implemented by the iMHEA network aim to achieve optimal complementarity with existing monitoring networks in the Andes^11^ and with national authorities of water, environment, hydrology and meteorology^25^. iMHEA's grassroots approach is based on the assumption that civil society-based institutions can contribute with local scale and headwater monitoring to cover remote areas, thus complementing other efforts of data collection. The high spatiotemporal resolution of these data is compatible with the generally long-term and low-spatial density of national networks^17,47^.

The monitoring system includes an institutional agreement between local users of land and water, development organisations or local government offices, academic institutions, and other monitoring networks. Local communities and institutions are responsible for sensor installation, operation, maintenance, and security and logistical tasks. Quality control and scientific rigour are achieved by research groups and universities who process and interpret the data. Such interaction, often understood as a form of citizen science^16–18^, has shown strong potential to tackle data scarcity in remote regions such as the tropical Andes. Observational data from experimental catchments have an essential value for hydrology and water resources management that increases with time^26^. As one of iMHEA's fundamental principle, this value is leveraged when information is shared at multiple levels for diverse audiences with the aim to inform decision-making processes more effectively. The network is interested in improving the management of water resources and hydrological ecosystem services in the Andean region, especially, to support the evaluation of the hydrological- and cost-effectiveness of watershed interventions. This institutional agreement provides an exemplary model to ensure the sustainability of monitoring networks in other contexts as well.

### Code availability

The codes used to process the iMHEA data are in the form of freely available MATLAB scripts (Data Citation 1). Calculations were done using MATLAB R2017a (version 9.2) under an Academic License provided by Imperial College London. Revised or improved versions of the code and adapted scripts for R and Python are also available in a GitHub repository^66^.

## Data Records

The list of the observatories used to generate the information in the present dataset is shown in Table 2. In this data descriptor, we present data from 9 sites of the iMHEA network, which account for 28 catchments comprising 67 rain gauges and 25 streamflow stations. Each site has a particular research question being addressed by the monitoring which generally fits under three broad categories: hydrological impacts of human activities, hydrological benefits of watershed interventions, and characterisation of hydrological processes. A summary of the dataset and monitored variables is provided in Table 3.

The data are intended to support decision making at multiple levels, both addressing relevant local questions and supporting regional analyses of water resources in the Andes. The hydrometeorological data as presented in this paper are archived at the public repository *Figshare* (Data Citation 1). A backup of the data, including the original raw files obtained from each particular monitoring sensor and expansions with further data updates, can be requested to the iMHEA network via the corresponding authors.

The file format is CSV. The naming convention for the sensor data is:

*iMHEA_<site code>_<catchment number>_<variable>_<sensor number>_raw.csv*

The naming convention for the compiled, processed catchment data is:

*iMHEA_<site code>_<catchment number>_<temporal resolution>_processed.csv*

The naming convention for the catchment properties and indices is:

*iMHEA_Indices_<index type>.csv*

The naming convention for the geographic data is:

*iMHEA_<site code>_<catchment number>_<geodata type>*

The site codes are defined by the three letters shown in Table 2. The variables are identified by two letters, where the first indicates the type of station (‘P’ for pluviometric, ‘H’ for hydrological). The second the type of sensor, (‘O’ for Onset HOBO, ‘D’ for Davis Instruments, ‘T’ for Texas Electronics) for precipitation, and (‘O’ for Onset HOBO, ‘S’ for Solinst, ‘D’ Schlumberger Diver, ‘W’ for Global Water, and ‘I’ for Instrumentation Northwest) for discharge measurement. Numbers identify catchments and sensor locations and are used to organise the matrices of physical and climatic characteristics and hydrological indices (Table 3). Geographic data include catchment delineation, sensor location, contour lines, drainage network, lithology, land cover, and digital elevation model.

## Technical Validation

### Regional representativeness and monitoring coverage

To maximise the regional representativeness of the tropical Andes and the potential for impact attribution, catchments were chosen to be hydrologically representative of a single land cover in the surrounding area^34^ (Fig. 3). Large catchments usually host various ecosystems or land uses, and therefore observational records may not capture the hydrological signals of individual management practices or land cover. This would make it difficult to attribute a streamflow response to the effect of a specific catchment intervention. As a result, the monitored catchments here are smaller than 20 km^2^ and host a homogeneous land use in at least 75% of their areas^25^. However, very small catchments (<0.2 km^2^) may have some experimental issues as well. For instance, in those with extensive wetland areas, subsurface water flow can become important^42^, increasing the risk of subsurface flow bypassing the flow gauging station. For this reason, our design avoids zero-order catchments^25^.

The number of stations necessary to approximate catchment rainfall input depends on the catchment area and the expected spatial rainfall variability therein^44^. A sub- or an over-estimation of total rainfall at a catchment level can lead to erroneous water balances and wrong conclusions about the impacts of different watershed interventions^25^. This is not a problem of the data themselves, but of their spatial extrapolation. Additionally, it has been found that tipping bucket rain gauges may underestimate total precipitation by around 15% for low intensity events in high Andean catchments^43^. Therefore, most of the iMHEA monitoring sites are equipped with 3 rain gauges at representative low, middle and high elevation points within the catchments^5^, or at least 2 rain gauges in the smaller areas (Table 4 (available online only)). This also allows validation and correction of measurements if one of the sensors fails, for example, due to battery or memory issues or inlet obstructions.

### Data collection and storage

As small mountain catchments feature a rapid hydrological response to precipitation events, the frequency of discharge data monitoring in the iMHEA sites is high (Table 4 (available online only)). The recommended minimum temporal interval for measuring water level is 15 min, but most of the stations record water levels every 5 minutes^25^. Tipping bucket rain gauges typically record data as tip time stamps (events), but a few stations use dataloggers that aggregate data at fixed intervals (Table 4 (available online only)). The exception is site CHA where rainfall and streamflow are both monitored at an interval of 30 min. The high temporal resolution allows identifying impacts on the short-term hydrological regulation, such as streamflow flashiness or hydrograph recession behaviour, that can be overlooked in aggregated indices^5^.

Although the most advanced sensors may have the capacity to store large amounts of data, batteries and data loggers have commonly a limited capacity to function for longer periods in harsh climatic conditions. These conditions and the remote locations reduce the possibility for regular sensor maintenance^25^. As a result, issues such as obstructions in the rain gauge orifices by forest litter or small animals may have occasionally occurred. To avoid the loss of important information, data are retrieved from each sensor approximately once a month.

Quality control of the generated data was performed immediately after the data have been collected in order to identify errors such as data outside the expected measurement range, negative water level values, or potential outliers such as extreme rainfall intensities. Data are curated thoroughly before their posterior processing and analysis as explained below. The high-resolution time series aggregated at different time intervals (5, 15, 60 min, or 1 day) represent a large amount of information in the long term (Figs 4a–c). However, it is imperative for impact assessment to store the finest resolution data to avoid losing important information that could be used in diverse studies and analyses^5,34^.

### Precipitation data uncertainty, quality control, and processing

We identify four main uncertainty sources in the monitoring and processing of rainfall data (Table 5): (i) equipment malfunction uncertainty, (ii) measurement errors, (iii) rainfall intensity interpolation, and (iv) spatial interpolation.

Equipment malfunction uncertainty can be investigated by comparing data with and without quality control^67^. Our rainfall data are first curated by differentiating between rainfall tip time stamps and software communication and functioning logs. The latter time stamps are still kept in the raw data files but flagged as ‘I’ when the sensor is launched, ‘D’ when data have been downloaded from the sensor, and ‘X’ when software logs have been removed from the rainfall count. Potentially erroneous tips (e.g, as a result of manipulating the rain gauge) have been flagged with an ‘X’. Other anomalies in the data, such as unrealistically high rainfall intensities, have been flagged in the raw files with the letter ‘P’.

Measurement uncertainty includes random errors such as turbulent airflow around the gauge^48,51^. Systematic errors, for example, underestimation of real rainfall due to wind loss, wetting loss, and splash-in/out, could theoretically be corrected when site-specific information is available^68^. We have found that the Onset HOBO rain gauges are prone to systematic anomaly, in which some time stamp intervals are exactly 1 s from the previous, which is the maximum time resolution of the data logger. Two tips of 0.2 mm with an interval of 1 s would represent a rainfall intensity of 720 mm hr^−1^. According to its manual^69^, the maximum rainfall intensity that this rain gauge can measure is 127 mm hr^−1^, and thus these immediate consecutive events where removed during the data depuration and processing. The standard deviation of the relative error in 1 hr rainfall rates can be approximated as a function of rainfall intensity^51^, which can then be used to estimate point measurement errors assuming a Gaussian distribution with zero mean^67^. By doing this, measurement uncertainty has been found to have only a small effect on the error standard deviation^67^.

Tipping bucket rain gauges aggregate rainfall data in the time domain, which complicates the estimation of rainfall intensities^68^. We use a composite cubic spline interpolation to generate a piecewise continuous curve that is thought to be the most accurate representation of rainfall rates^50,70^ (Figs 4d and e), but this is based on several assumptions^43,49–51^. Four threshold intensities were defined: (i) a minimum intensity to separate rainfall events, which depends on the rainfall structure of the studied region (here, 0.2 mm hr^−1^)^43^; (ii) a maximum intensity to merge consecutive tips, which is mainly to avoid issues when concatenating consecutive high- and low-intensity intervals (here, 127 mm hr^−1^)^69^; (iii) a mean intensity to distribute single tips due to the lack of information to interpolate them (here, 3 mm hr^−1^)^50^; and, (iv) a low threshold intensity above which data are accepted to avoid having extremely low rainfall rates (here, 0.1 mm hr^−1^)^50^. To estimate the ‘real’ initial and final extremes of rainfall events, we assumed that they occur at half the rainfall rate of the two extreme rainfall tips and considered half partial tip remaining in the bucket from the previous event^49^. The first derivatives are then set to zero at these new defined extremes when applying the cubic spline interpolation. This improves the rainfall rate interpolation at the extremes of rainfall events and is otherwise equivalent to not estimating the initial and end points and setting the second derivatives to zero at the extremes.

Data were aggregated at 1 min resolution using tip counting before proceeding with the interpolation^50^. In the case that independent rainfall events had only 2 data points, a linear interpolation^51^ was used instead. Mass-conservation and discontinuities were checked in the interpolated data for each independent rainfall event^50^: (i) when the estimated rainfall rates were negative (produced by a rapid change in the spline slopes between high and low rainfall rates), they were replaced by zero; (ii) rates that were between 0 and the low threshold intensity are replaced by this low threshold intensity; (iii) the resulting bias between the original and the interpolated rainfall totals was corrected by re-scaling the remaining intensities. Then, the cumulative rainfall curve was reconstructed by consecutively adding the corrected rainfall rates. In the rare cases when the bias after the interpolation was unacceptably large (here, >25%, although some studies use a tolerance of as much as 50% (ref. 50)), the cubic spline interpolation was discarded and a linear interpolation was applied instead.

Spatial interpolation errors affect the calculation of catchment average rainfall from the point measurements and depend on rain gauge density, network design, and rainfall spatial variability, which in turn is affected by topography, rainfall intensity, and storm type^44,67^. Interpolation errors in large catchments can be investigated by subsampling from the rain gauge network^67^. Here, we constrain spatial uncertainty by using at least two rain gauges in catchments much smaller than 20 km^2^, which is well above the WMO-recommended station density of 1 gauge 250 km^−2^ for mountain environments^47^. Data gaps identified in the time series are flagged with a letter ‘V’ in the raw files, and monitored periods and percentages of data gaps for each sensor are shown in Table 4 (available online only). Rainfall data within each catchment were contrasted between the several rain gauges using double mass plots. We use the double mass plots also to fill data gaps in the rain gauges, but only when the regression coefficient (R^2^) was larger than 0.99. To calculate catchment average rainfall, we used a simple average of rain gauge data within each catchment. Rainfall data between paired catchments were used to fill any remaining gaps, again only when R^2^>0.99.

### Streamflow data uncertainty, quality control, and processing

Monitoring and processing of streamflow data are affected by several sources of uncertainty (Table 5), from which we consider the most important to be: (i) uncertainty in the measurement of water level and discharge, (ii) rating curve calibration and extrapolation uncertainty (iii) time step used in the calculation of hydrological indices, (iv) seasonality in the hydrological response, and (v) hydrological index calculation method.

Data collected using pressure transducers need temperature and barometric pressure compensation. Daily variations in surface water level have been observed in several studies^71–74^. While this phenomenon has been attributed to a set of confounding factors^75^, including evapotranspiration of riparian vegetation^76^, it may also be the consequence of an inappropriate monitoring design and operation^73^, for instance, external sensors, loggers, or cables directly exposed to solar radiation or precipitation. To minimise these issues, the barometric and submerged sensors of the absolute pressure transducer systems were installed in temperature conditions that were as similar and as buffered as possible. Vented tube sensors, in principle, do not require additional barometric compensation^74^; nonetheless, when using them, we ensure the vented tube was free of obstructions, dry and isolated from direct solar radiation to avoid potential issues as recommended^77–79^. Uncertainty in the measurement of water level time series can be approximated by sampling from a uniform distribution using the nominal sensor accuracy as the estimated error (Table 6). Previous studies have used a uniform distribution with a mid-range value of ±5 mm (ref. 80) to investigate the propagation of uncertainty from discharge data to the calculation of hydrological indices^67,81^. Discharge uncertainty is typically larger than water level uncertainty and can be difficult to estimate especially during high-flow gaugings^67^.

Water level data were validated and corrected using manual observations during each site visit. The water depth over the weir was measured manually with 1 mm precision and then used to compensate differences with respect to the values collected by the automatic water level sensors. The same convention as for rainfall data was used for water level and discharge data, flagging data in the raw files (i.e., letters ‘I’, ‘D’, ‘X’, ‘P’, and ‘V’). Water level data gaps were only filled for short periods in which the sensors were stopped for data retrieving, which were generally less than a few hours (Table 4 (available online only)). Other data gaps in water level or discharge data were not filled to avoid the manipulation of hydrological data that could result in erroneous attributions of streamflow responses. For instance, a non-linear impact of livestock grazing on the hydrological response time and flow recession curve can be observed in Fig. 4f. A linear interpolation of measurement gaps could affect this non-linear impact and potentially hinder the attribution of observed differences in the hydrological response.

The approximation of the true stage–discharge relation by means of a rating curve is usually a dominant source of uncertainty^67,82^, especially when this relationship changes over time. Processes that affect the validity of the rating curve include changes in roughness, vegetation growth, upstream sediment deposition and erosion, out-of-bank flow ranges, and unsteady flows or backwater effects^81,83^. In the monitored iMHEA catchments, flows are contained within a weir, which constrains the rating curve uncertainty and allows application of a theoretical relation derived from hydraulic principles (the Kindsvater–Shen equation^56^). Only in a few catchments was this relation compared to direct flow observations. Table 6 shows the weir dimensions used for the Kindsvater–Shen equation in each catchment. Several iMHEA sites have started the task of deriving specific stage-discharge curves to improve the accuracy in the calculation of streamflow data from water levels. In the literature, different parameterisations have been tried, with polynomial functions performing slightly better than power functions^83^. A previous study investigating the use of stage-discharge rating curves in non-ideal conditions for short-term projects, in which measurements were generally gathered by non-specialists, found large uncertainties in the calculation of cumulative annual discharge between −13 and +14% (ref. 84).

The extremes of the rating curve tend to be more uncertain because of the lower number of low and high flows available as calibration points. Previous studies on quantitative analysis of discharge estimation^67,81–83^ have found that high flow extrapolation is the dominant source of uncertainty as the conditions that control the stage-discharge relationship can be different at water levels much higher than average. These errors can introduce much larger uncertainty than river flow measurements and seasonal changes in roughness. Additionally, being the non-linear least squares method the standard technique to calibrate rating curves, its lack of flexibility may introduce heteroscedasticity that needs to be accounted for^85^. In the case of unsteady flow occurrence, a dynamic rating curve approach can be used to provide an indirect discharge measurement based on simultaneous water level measurements at adjacent cross sections^86^, but this has not been implemented in our monitoring protocol^25^ and is still not common in national level hydrometeorological monitoring elsewhere^47^.

Uncertainty in the calculation of hydrological indices results from the time discretisation of the flow data. This can be investigated by comparing indices calculated at different time steps (e.g. hourly and daily). For instance, using these hydrometeorological data to evaluate the effects of land-use changes, Ochoa-Tocachi *et al.* (2016, ref. 5) have found that impacts on the short-term hydrological regulation can remain unnoticed on daily aggregated indices but become evident when using high-resolution time series. Other sources of uncertainty arise from seasonal variations in the hydrological response that can provide evidence of catchment intervention impacts^38^ or, in contrast, obscure statistical differences between catchments and between different monitoring periods^34^. The calculation method of particular hydrological indices can also introduce uncertainty in the assessment of catchment interventions. For instance, the baseflow index and its correspondent recession constant were calculated here using two different methods, one proposed by the UK Flood Estimation Handbook^64^ and the other is a two-parameter algorithm^65^ fitted subjectively. The shapes of the derived baseflow time series are similar, and the general trends observed between catchments are consistent. However, the different absolute values can lead to diverse interpretations of how groundwater dominated the catchments are. Lastly, if catchment data are pooled, for example in hydrological modelling or regionalisation, then the propagation of uncertainty from rainfall and streamflow data to any derived synthetic time series, model parameter, or hydrological index, as well as the uncertainty in the implemented methods and model structural errors, need to be considered and quantified^81,87^.

## Usage Notes

The iMHEA dataset is intended to analyse the hydrological impacts of human activities in Andean catchments (e.g., land use change), to support the evaluation of hydrological benefits of watershed interventions (e.g., restoration strategies), or to increase understanding of hydrological processes of natural ecosystems and green infrastructure (e.g., water harvesting techniques). Recent applications of these data include evidence-based, robust predictions and cost-benefit comparisons of the effectiveness of different watershed interventions to support institutions in their ex-ante planning, implementation, and ex-post evaluation^5,88^. Such analyses can be extended to hydrological model calibration and regional extrapolation to make predictions in ungauged basins^34^.

We do not consider the data to be suitable for trend analysis because of the short length of the time series. The value of the ‘trading-space-for-time approach’ relies on the comparison of climate conditions and hydrological responses between the paired catchments^5,38^. However, users need to be aware that catchments are unique^89^ and their physical characteristics are inherently different, and thus land use and land cover are the main but not the only variance between them. An added value of the monitoring network design is the ability to compare and regionalise results by pooling the catchments together in a regional impact model^34^. In this case, users need to be cautious of the different monitoring periods of sites and catchments which may be influenced by particular regional hydrometeorological drivers.

The iMHEA sites are also available for the implementation of external research projects that can contribute to generate better and more detailed understanding of hydrological processes and impacts of human activities on water quantity and quality, sediment transport, and other ecosystem services. The long-term sustainability in the monitoring of experimental catchments will allow a deeper understanding of seasonality, natural variability, environmental changes, and extreme events such as drought and flooding^26^. The sites have been defined based on their socioeconomic relevance for local and regional stakeholders and can benefit from extending the monitoring to other variables or methods of interest. We invite researchers worldwide to make use of this dataset, and to engage in and complement the monitoring of these and new iMHEA sites.

The data are freely available under the Creative Commons Licence: CC BY 4.0.

## Additional information

**How to cite this article:** Ochoa-Tocachi B. F. *et al.* High-resolution hydrometeorological data from a network of headwater catchments in the tropical Andes. *Sci. Data* 5:180080 doi: 10.1038/sdata.2018.80 (2018).

**Publisher’s note:** Springer Nature remains neutral with regard to jurisdictional claims in published maps and institutional affiliations.

## Supplementary Material



## Figures and Tables

**Table 1:** Description of the monitored catchments. For a reference of site codes, see Table 2.

**Code**	**Biome**	**Altitude m a.s.l.**	**Area km**^**2**^	**Shape**	**Slope**	**Soils**	**Land use**	**Land cover % approx.**
LLO_01	Páramo	3825–4700	1.79	Stretched oval	Strongly uneven	Andosol	Grazing, burning	Tussock grass (90), shrubs (10)
LLO_02	Páramo	4088–4680	2.21	Stretched oval	Uneven	Andosol, Histosol	Grazing, restoration	Tussock grass (70), native forest (10), wetlands (20)
JTU_01	Páramo	4075–4225	0.65	Oval	Uneven	Andosol	Grazing	Tussock grass (100)
JTU_02	Páramo	4085–4322	2.42	Oval	Uneven	Andosol	Grazing	Tussock grass (100)
JTU_03	Páramo	4144–4500	2.25	Circular oval	Uneven	Andosol, Histosol	Natural	Tussock grass (80), shrubs (20)
JTU_04^a^	Páramo	3990–4530	16.05	Stretched oval	Uneven	Andosol, Histosol	Grazing, natural, restoration	Tussock grass (70), shrubs (10), wetlands (5), nude soil (15)
PAU_01	Páramo	3665–4100	2.63	Circular oval	Uneven	Andosol	Natural	Tussock grass (100)
PAU_02	Páramo	2970–3810	1.00	Oval	Strongly uneven	Andosol, Histosol	Natural, grazing	Tussock grass (80), native forest (20)
PAU_03	Páramo	3245–3680	0.59	Circular oval	Strongly uneven	Andosol, Histosol	Afforestation	Tussock grass (10), pine plantation (90)
PAU_04	Páramo	3560–3721	1.55	Circular oval	Uneven	Andosol	Cultivation, grazing	Tussock grass (70), crops (30)
PAU_05^b^	Páramo	3300–3500	-	-	-	Andosol, Cambisol	Natural	Tussock grass, shrubs
PIU_01	Páramo	3112–3900	6.60	Circular oval	Uneven	Andosol, Histosol	Natural	Tussock grass (75), native forest (15), lagoon (10)
PIU_02	Páramo	3245–3610	0.95	Circular oval	Strongly uneven	Andosol, Histosol	Grazing	Tussock grass (75), nude rock (15), lagoon (10)
PIU_03	Páramo	3425–3860	1.31	Circular oval	Strongly uneven	Andosol, Histosol	Grazing	Tussock grass (90), lagoon (10)
PIU_04	Forest	2682–3408	2.32	Oval	Strongly uneven	Andosol, Cambisol	Natural forest	Grass (20), native forest (80)
PIU_05^b^	Forest	1972–2176	-	-	-	Andosol, Cambisol	Cultivation, forest	Native forest, crops
PIU_06^b^	Forest	2782–3078	-	-	-	Andosol, Cambisol	Cultivation, forest	Native forest, crops
PIU_07	Dry puna	3110–3660	7.80	Oval	Uneven	Andosol	Grazing, cultivation	Tussock grass (45), shrubs (20), crops (35)
CHA_01	Jalca	2940–3200	0.95	Oval	Uneven	Andosol, Inceptisol	Afforestation	Tussock grass (20), pine plantation (80)
CHA_02	Jalca	3000–3450	1.63	Oval	Uneven	Andosol, Inceptisol	Natural	Tussock grass (90), native forest (10)
HUA_01	Humid puna	4280–4840	4.22	CO	Uneven	Andosol, Histosol	Natural	Tussock grass (60), nude rock (25), wetlands (15)
HUA_02^c^	Humid puna	4235–4725	2.38	Oval	Uneven	Andosol, Histosol	Grazing	Tussock grass (55), nude rock (30), wetlands (15)
HMT_01	Dry puna	4025–4542	2.09	Oval	Uneven	Leptosol, Inceptisol	Grazing	Grass (75), nude soil (15), shrubs (10)
HMT_02	Dry puna	3988–4532	1.69	Oval	Strongly uneven	Leptosol, Inceptisol	Grazing	Grass (85), nude soil (10), shrubs (5)
TAM_01	Humid puna	3835–4026	0.82	Oval	Uneven	Leptosol, Inceptisol	Afforestation, grazing	Grass (80), pine plantation (20)
TAM_02	Humid puna	3650–4360	1.67	Circular oval	Strongly uneven	Leptosol, Inceptisol	Natural, forest	Grass (60), native forest (40)
TIQ_01	Humid puna	4140–4353	0.69	Oval	Uneven	Leptosol, Inceptisol	Cultivation, grazing	Grass and crops (70), nude soil (30)
TIQ_02	Humid puna	4182–4489	1.73	Stretched oval	Uneven	Leptosol, Inceptisol	Natural	Tussock grass (90), nude soil (5), lagoon (5)

^a^JTU_04 is a large catchment containing the other three JTU catchments.

^b^PAU_05, PIU_05, and PIU_06 are monitored for rainfall only and, as they lack streamflow data, their areas are not defined.

^c^The discharge station in HUA_02 was initially located downstream from its current position defining an area of 2.71 km^2^; data from this early period have been normalised during the data processing to use only the current area shown here.

**Table 2:** Summary of the iMHEA monitoring sites presented in the dataset (Data Citation 1).

**Site code, name, country**	**Biome types**	**Monitoring design**	**Monitoring period**	**Research questions / partners**
LLO, Lloa, Ecuador	Páramo	2C / 4 P / 2H	01/2013–01/2017	Impacts of grazing, burning, and deep soil hydrological connectivity / Fondo para la Protección del Agua (FONAG); Junta Parroquial de Lloa (local government)
JTU, Jatunhuayco, Ecuador	Páramo	4C / 8 P / 4H	11/2013–01/2017	Hydrological benefits of restoration strategies / FONAG; Escuela Politécnica Nacional de Quito
PAU, Paute, Ecuador	Páramo	5C / 13 P / 4H	05/2001–07/2007	Hydrological impacts of afforestation and cultivation in humid páramo / Universidad de Cuenca; Empresa pública municipal de telecomunicaciones, agua potable, alcantarillado y saneamiento de Cuenca (ETAPA EP)
PIU, Piura, Peru	Páramo; Forest	7C / 20 P / 5H	04/2013–01/2017	Benefits of conservation and impacts of land use for downstream users / Nature and Culture International (NCI)
CHA, Chachapoyas, Peru	Jalca	2C / 2 P / 2H	09/2010–12/2015	Impacts of pine afforestation / Asociación Peruana para la Conservación de la Naturaleza (APECO); Universidad Nacional de Colombia.
HUA, Huaraz, Peru	Humid puna	2C / 6 P / 4H	02/2011–09/2014	Hydrological benefits of pasture restoration and impacts of grazing / The Mountain Institute; Universidad Nacional Santiago Antúnez de Mayolo
HMT, Huamantanga, Peru	Dry puna	2C / 4 P / 2H	06/2014–01/2017	Hydrological benefits of pasture restoration, impacts of grazing, and hydrological characterisation of water harvesting techniques / CONDESAN; Universidad Nacional Agraria La Molina; Imperial College London
TAM, Tambobamba, Peru	Humid puna	2C / 6 P / 2H	04/2012–04/2013	Impacts of pine afforestation and infiltration trenches / Municipalidad Provincial de Cotabambas
TIQ, Tiquipaya, Bolivia	Humid puna	2C / 4 P / 2H	02/2013–01/2016	Impacts of cultivation and grazing / Laboratorio de Hidráulica – Universidad Mayor de San Simón
Letters ‘C’, ‘P’, and ‘H’ in column 3 indicate the number of catchments, rain gauges, and streamflow stations for each site, respectively. Site locations are shown in Fig. 1.				

**Table 3:** Overview of the variables included in the dataset (Data Citation 1).

**Variable Name**	**Raw data**	**Methods**	**Processed data**	**Details**
Precipitation	One file per sensor, geopositionRain gauge tips at 0.254, 0.2, or 0.1 mm; or cumulative values at 5 or 30 min resolution	Tip depuration,Cubic spline interpolation on the cumulative rainfall curve,Rainfall accumulation,Data gap filling	One file per catchment,Precipitation time series at maximum temporal resolution matching discharge, and at 1 hr and 1 day resolutions	Units: rainfall (mm).Some stations (JTU, CHA) lack rain gauge tip data as the logger was programmed to store cumulative values only
Streamflow	One file per sensor, geoposition,Water level instantaneous measurements at 5, 15, or 30 min resolution	Water level validation,Discharge calculation,Restricted data gap filling	One file per catchment,Streamflow time series at maximum temporal resolution, and at 1 hr and 1 day resolutions	Units: water level (cm), streamflow (l s^−1^ km^−2^).Weir dimensions used to convert water level to discharge for each station in Table 6
Catchment characteristics	Geographic information data	Calculations using Geographic Information Systems software and custom scripts	Matrix of physical characteristics (rows) for each catchment (columns)Maps of each catchment or pair of catchments	Categories: shape, drainage, elevation, topography, subsurface, climate, rainfall intensity, land cover and land use
Hydrological indices		Hydrological index calculation based on custom scripts, averaged over their monitoring periods	Matrix of hydrological indices (rows) for each catchment (columns)	Categories: water yield, hydrological regulation, water balance, magnitude, frequency, duration, timing, flashiness

**Table 4:** Detail of sensors monitored by the iMHEA network (Data Citation 1). *JTU_04 is a large catchment containing the other three JTU catchments.

**Country**	**Site**	**Sensor**	**Longitude**	**Latitude**	**Altitude m a.s.l.**	**Variable**	**Time scale**	**Monitoring period**	**Gaps %**	**Catchment**	**Time series length**	**Gaps %**
ECU	LLO	LLO_01_PO_01	-78.58981817	−0.19583548	3897.60	Rainfall	0.2 mm	10/01/2013 - 13/01/2017	29.25	LLO_01	10/01/2013 - 13/01/2017	0.01
		LLO_01_PO_02	−78.58760121	−0.19010428	4076.89	Rainfall	0.2 mm	10/01/2013 - 13/01/2017	4.44			
		LLO_01_HI_01	−78.59048236	−0.19747168	3824.79	Water level	5 min	10/01/2013 - 13/01/2017	0.78			0.79
		LLO_02_PO_01	−78.58554572	−0.18543071	4144.20	Rainfall	0.2 mm	10/01/2013 - 13/01/2017	4.36	LLO_02	10/01/2013 - 13/01/2017	0.01
		LLO_02_PO_02	−78.58607629	−0.17923891	4219.80	Rainfall	0.2 mm	10/01/2013 - 13/01/2017	1.64			
		LLO_02_HI_01	−78.58383932	−0.18858516	4088.00	Water level	5 min	10/01/2013 - 13/01/2017	26.06			26.07
	JTU	JTU_01_PT_01	−78.24061389	−0.48910556	4104.00	Rainfall	0.1 mm	20/11/2013 - 17/11/2017	9.26	JTU_01	14/11/2013 - 17/01/2017	3.36
		JTU_01_PT_02	−78.24419722	−0.48681111	4148.00	Rainfall	0.1 mm	20/11/2013 - 17/11/2017	10.90			
		JTU_01_HI_01	−78.23934722	−0.48841389	4075.00	Water level	5 min	14/11/2013 - 17/11/2017	0.56			0.56
		JTU_02_PT_01	−78.24175833	−0.48106389	4110.00	Rainfall	5 min	19/01/2014 - 18/01/2017	2.29	JTU_02	15/11/2013 - 18/01/2017	5.61
		JTU_02_PT_02	−78.24141111	−0.47195278	4271.00	Rainfall	5 min	19/01/2014 - 17/01/2017	0.00			
		JTU_02_HI_01	−78.23984444	−0.48200278	4085.00	Water level	5 min	15/11/2013 - 18/01/2017	0.18			0.18
		JTU_03_PT_01	−78.23153056	−0.46802778	4203.00	Rainfall	0.1 mm	19/11/2013 - 20/01/2017	3.63	JTU_03	13/11/2013 - 20/01/2017	0.51
		JTU_03_PT_02	−78.22835000	−0.46894722	4231.00	Rainfall	5 min	05/01/2014 - 16/01/2017	0.00			
		JTU_03_HI_01	−78.23121389	−0.46932778	4144.00	Water level	5 min	13/11/2013 - 20/01/2017	0.82			0.82
		JTU_04_PT_01	−78.23745556	−0.50437500	4023.00	Rainfall	0.1 mm	19/11/2013 - 16/01/2017	9.66	JTU_04*	19/11/2013 - 20/01/2017	0.00
		JTU_04_PT_02	−78.21156667	−0.46619444	4289.00	Rainfall	0.1 mm	19/11/2013 - 16/01/2017	5.64			
		JTU_04_HI_01	−78.23936111	−0.50360833	4103.00	Water level	5 min	16/01/2014 - 11/02/2016	0.14			34.82
	PAU	PAU_01_PD_01	−79.02911100	−2.66698000	3694.00	Rainfall	0.2 mm	24/05/2001 - 17/06/2005	5.77	PAU_01	24/05/2001 - 16/08/2005	3.88
		PAU_01_PD_02	−79.02651371	−2.66041178	3875.00	Rainfall	0.2 mm	24/05/2001 - 17/06/2005	0.00			
		PAU_01_PD_03	−79.02033880	−2.65920832	3824.00	Rainfall	0.2 mm	24/05/2001 - 17/06/2005	5.11			
		PAU_01_HW_01	−79.02996318	−2.66801240	3675.00	Water level	15 min	04/09/2001 - 16/08/2005	15.97			21.60
		PAU_04_PD_01	−-78.99521700	--2.70948400	3562.00	Rainfall	0.2 mm	05/02/2002 - 14/10/2003	0.00	PAU_04	27/10/2001 - 14/10/2003	0.00
		PAU_04_PD_02	−79.00565763	−2.69745775	3671.00	Rainfall	0.2 mm	27/10/2001 - 18/06/2003	22.32			
		PAU_04_PD_03	−78.99200155	−2.70749840	3653.00	Rainfall	0.2 mm	27/10/2001 - 14/10/2003	38.03			
		PAU_04_HW_01	−78.99523522	−2.70942059	3562.00	Water level	15 min	29/10/2001 - 29/09/2003	11.29			13.33
		PAU_02_PD_01	−79.12021708	−2.85740814	3621.70	Rainfall	0.254 mm	29/05/2004 - 31/07/2007	0.00	PAU_02	29/02/2004 - 31/07/2007	7.19
		PAU_02_PD_02	−79.12400918	−2.85368897	3438.66	Rainfall	0.254 mm	29/05/2004 - 31/07/2007	0.00			
		PAU_02_HW_01	−79.11650102	−2.84755507	2976.00	Water level	15 min	29/02/2004 - 27/06/2007	0.00			2.73
		PAU_03_PD_01	−79.10916022	−2.86014775	3255.00	Rainfall	0.254 mm	29/05/2004 - 03/10/2004	12.00	PAU_03	29/05/2004 - 31/07/2007	0.00
		PAU_03_PD_02	−79.11459178	−2.85476759	3375.71	Rainfall	0.254 mm	29/05/2004 - 31/07/2007	0.00			
		PAU_03_HW_01	−79.10916022	−2.86014775	3255.00	Water level	15 min	29/05/2004 - 27/06/2007	26.58			28.74
		PAU_05_PD_01	−78.75328100	−2.57024600	3330.00	Rainfall	0.2 mm	27/02/2002 - 31/05/2005	0.00	PAU_05	27/02/2002 - 31/05/2005	0.00
		PAU_05_PD_02	−78.75508500	−2.56123600	3374.00	Rainfall	0.2 mm	05/03/2002 - 23/04/2005	5.40			
		PAU_05_PD_03	−78.74953600	−2.55757400	3438.00	Rainfall	0.2 mm	05/03/2002 - 22/10/2004	0.00			
PER	PIU	PIU_01_PO_01	−79.48006182	−4.97327714	3202.10	Rainfall	0.2 mm	06/07/2013 - 11/05/2016	0.00	PIU_01	05/07/2013 - 08/03/2017	0.03
		PIU_01_PO_02	−79.47514373	−4.97478076	3227.01	Rainfall	0.2 mm	06/07/2013 - 08/03/2017	8.75			
		PIU_01_PO_03	−79.46580994	−4.97629635	3263.06	Rainfall	0.2 mm	06/07/2013 - 08/03/2017	15.46			
		PIU_01_HI_01	−79.48005264	−4.97325014	3192.94	Water level	5 min	05/07/2013 - 12/12/2015	0.00			33.69
		PIU_02_PO_01	−79.48082953	−4.94313871	3258.45	Rainfall	0.2 mm	06/07/2013 - 07/12/2016	0.00	PIU_02	06/07/2013 - 07/12/2016	0.00
		PIU_02_PO_02	−79.47937127	−4.94158826	3273.63	Rainfall	0.2 mm	06/07/2013 - 23/04/2014	44.31			
		PIU_02_PO_03	−79.47303904	−4.94005271	3289.25	Rainfall	0.2 mm	06/07/2013 - 07/08/2015	21.64			
		PIU_02_PO_04	−79.47740253	−4.94436147	3289.25	Rainfall	0.2 mm	23/04/2014 - 07/12/2016	0.00			
		PIU_02_HI_01	−79.48117027	−4.94299210	3244.79	Water level	5 min	07/07/2013 - 13/12/2015	24.77			46.50
		PIU_03_PO_01	−79.46073672	−4.73858660	3500.50	Rainfall	0.2 mm	11/04/2013 - 09/04/2014	30.56	PIU_03	11/04/2013 - 10/08/2016	7.15
		PIU_03_PO_02	−79.45942327	−4.73845078	3526.21	Rainfall	0.2 mm	11/04/2013 - 17/08/2014	31.83			
		PIU_03_PO_03	−79.45702546	−4.73885411	3473.34	Rainfall	0.2 mm	11/04/2013 - 10/08/2016	9.12			
		PIU_03_PO_04	−79.45840993	−4.74304826	3644.70	Rainfall	0.2 mm	09/04/2014 - 10/08/2016	0.00			
		PIU_03_HI_01	−79.46091715	−4.73868464	3495.21	Water level	5 min	23/06/2013 - 04/12/2013	55.53			94.01
		PIU_04_PO_01	−79.45941222	−4.70158815	2747.07	Rainfall	0.2 mm	11/07/2013 - 04/05/2016	0.00	PIU_04	23/06/2013 - 20/01/2017	15.86
		PIU_04_PO_02	−79.46350783	−4.70523279	2925.64	Rainfall	0.2 mm	11/07/2013 - 15/07/2016	8.71			
		PIU_04_PO_03	−79.46412158	−4.71265185	3113.81	Rainfall	0.2 mm	11/07/2013 - 11/08/2016	40.98			
		PIU_04_HI_01	−79.45925630	−4.70110210	2727.37	Water level	5 min	23/06/2013 - 20/01/2017	16.62			16.62
		PIU_05_PO_01	−79.66289905	−5.00413357	1972.00	Rainfall	0.2 mm	21/08/2014 - 09/12/2016	19.09	PIU_05	21/08/2014 - 09/12/2016	19.09
		PIU_06_PO_01	−79.64667662	−5.04818063	2797.06	Rainfall	0.2 mm	19/02/2014 - 11/08/2016	15.22	PIU_06	19/02/2014 - 11/08/2016	1.28
		PIU_06_PO_02	−79.65544300	−5.05061300	3078.00	Rainfall	0.2 mm	19/02/2014 - 14/12/2015	23.05			
		PIU_06_PO_03	−79.65020646	−5.04635189	2830.47	Rainfall	0.2 mm	18/06/2015 - 13/05/2016	3.87			
		PIU_07_PO_03	−79.82075251	−4.97860295	3117.66	Rainfall	0.2 mm	11/07/2013 - 28/08/2014	0.00	PIU_07	11/07/2013 - 16/01/2017	22.93
		PIU_07_PO_02	−79.82033172	−4.98006645	3121.26	Rainfall	0.2 mm	11/07/2013 - 16/01/2017	31.11			
		PIU_07_HI_01	−79.82085774	−4.97824158	3120.06	Water level	5 min	16/07/2013 - 15/01/2015	0.00			57.33
	CHA	CHA_01_PT_01	−77.78205513	−6.33685022	3112.00	Rainfall	30 min	04/09/2010 - 07/12/2015	0.00	CHA_01	04/09/2010 - 07/12/2015	0.00
		CHA_01_HS_01	−77.78134038	−6.34686083	3030.00	Water level	30 min	17/07/2012 - 19/10/2013	0.00			76.10
		CHA_02_PT_01	−77.83107400	−6.33465000	3142.00	Rainfall	30 min	03/09/2010 - 07/12/2015	0.00	CHA_02	03/09/2010 - 07/12/2015	0.00
		CHA_02_HS_01	−77.83341190	−6.34503339	2934.00	Water level	30 min	15/07/2012 - 24/01/2014	0.00			70.95
	HUA	HUA_01_PD_01	−77.35213200	−9.67455900	4417.00	Rainfall	0.2 mm	27/02/2011 - 20/06/2014	26.99	HUA_01	27/02/2011 - 27/11/2014	13.89
		HUA_01_PD_02	−77.34960300	−9.66992000	4451.00	Rainfall	0.2 mm	27/02/2011 - 20/06/2014	7.77			
		HUA_01_PD_03	−77.34229900	−9.67209900	4484.00	Rainfall	0.2 mm	27/02/2011 - 20/06/2014	37.89			
		HUA_01_HD_01	−77.35704800	−9.67302500	4306.00	Water level	5 min	18/04/2011 - 10/09/2012	24.52			14.07
		HUA_01_HD_02	−77.35704800	−9.67302500	4306.00	Water level	5 min	27/09/2012 - 27/11/2014	0.02			
		HUA_02_PD_01	−77.35135100	−9.69735700	4424.00	Rainfall	0.2 mm	25/02/2011 - 20/06/2014	10.24	HUA_02	25/02/2011 - 05/09/2014	8.30
		HUA_02_PD_02	−77.34975200	−9.69157100	4425.00	Rainfall	0.2 mm	25/02/2011 - 20/06/2014	2.48			
		HUA_02_PD_03	−77.34348700	−9.69281700	4480.00	Rainfall	0.2 mm	25/02/2011 - 20/06/2014	41.76			
		HUA_02_HD_01	−77.35120600	−9.69425900	4356.00	Water level	5 min	18/04/2011 - 10/09/2012	24.52			15.53
		HUA_02_HD_02	−77.35120600	−9.69425900	4356.00	Water level	5 min	16/09/2012 - 05/09/2014	2.34			
	HMT	HMT_01_PO_01	−76.73923100	−11.46868700	4185.91	Rainfall	0.2 mm	07/07/2014 - 01/10/2015	0.00	HMT_01	28/06/2014 - 04/01/2017	21.83
		HMT_01_PO_02	−76.73274300	−11.46094700	4397.40	Rainfall	0.2 mm	07/07/2014 - 04/01/2017	23.74			
		HMT_01_HI_01	−76.74202900	−11.47246900	4001.58	Water level	5 min	28/06/2014 - 04/01/2017	0.77			0.77
		HMT_02_PO_01	−76.71868300	−11.47377800	4110.69	Rainfall	0.2 mm	26/06/2014 - 04/01/2017	0.00	HMT_02	26/06/2014 - 04/01/2017	0.00
		HMT_02_PO_02	−76.72767100	−11.46647700	4347.65	Rainfall	0.2 mm	07/07/2014 - 03/03/2016	18.79			
		HMT_02_HI_01	−76.71787000	−11.47657500	3913.86	Water level	5 min	26/06/2014 - 04/01/2017	0.00			0.00
	TAM	TAM_01_PO_01	−72.19776384	−14.00225577	4015.00	Rainfall	0.2 mm	12/04/2012 - 02/01/2013	0.00	TAM_01	12/04/2012 - 02/01/2013	0.00
		TAM_01_PO_02	−72.19286754	−14.00325589	3939.00	Rainfall	0.2 mm	13/04/2012 - 19/09/2012	0.00			
		TAM_01_PO_03	−72.19044548	−14.00236974	3872.00	Rainfall	0.2 mm	13/04/2012 - 19/09/2012	22.56			
		TAM_01_HO_01	−72.19014354	−14.00133656	3851.00	Water level	5 min	13/04/2012 - 30/08/2012	0.00			47.70
		TAM_02_PO_01	−72.17280693	−13.98711691	4142.00	Rainfall	0.2 mm	12/04/2012 - 11/12/2012	0.00	TAM_02	12/04/2012 - 16/04/2013	0.00
		TAM_02_PO_02	−72.17958919	−13.98389006	3940.00	Rainfall	0.2 mm	12/04/2012 - 22/01/2013	12.70			
		TAM_02_PO_03	−72.18733420	−13.98438643	3713.00	Rainfall	0.2 mm	12/04/2012 - 16/04/2013	0.00			
		TAM_02_HO_01	−72.18822609	−13.98406257	3708.00	Water level	5 min	12/04/2012 - 31/08/2012	0.00			61.91
BOL	TIQ	TIQ_01_PO_01	−66.24166695	−17.26002013	4306.00	Rainfall	0.2 mm	02/04/2013 - 25/01/2016	0.00	TIQ_01	02/04/2013 - 25/01/2016	0.00
		TIQ_01_PO_02	−66.23504520	−17.25657872	4206.00	Rainfall	0.2 mm	02/04/2013 - 25/01/2016	0.00			
		TIQ_01_HD_01	−66.23321600	−17.25319416	4140.00	Water level	5 min	20/03/2014 - 24/08/2015	0.00			49.19
		TIQ_02_PO_01	−66.21142589	−17.21473015	4357.00	Rainfall	0.2 mm	02/04/2013 - 25/01/2016	1.66	TIQ_02	18/02/2013 - 25/01/2016	4.01
		TIQ_02_PO_02	−66.21660437	−17.21528045	4316.00	Rainfall	0.2 mm	02/04/2013 - 17/12/2014	14.42			
		TIQ_02_HD_01	−66.22075352	−17.22057549	4182.00	Water level	5 min	18/02/2013 - 25/01/2016	0.35			0.00

**Table 5:** Considered sources of uncertainty and investigation methods, following Westerberg and McMillan (2015, ref. 67).

**Variable**	**Uncertainty sources**	**Investigation method**	**Reference**
Precipitation	Equipment malfunction	Rainfall data with and without quality control	^90^
	Point measurement error	Normal distribution with standard deviation as a function of rain rate	^48,51^
	Rainfall intensity interpolation method, rainfall event definition	Composite cubic spline interpolation, threshold intensities, and bias correction	^43,49–51,70^
	Spatial interpolation uncertainty	Subsampling from the available rain gauges in the network	^67^
Streamflow	Water level measurement uncertainty, barometric and temperature pressure compensation	Sampling from uniform or normal distributions with standard error of ±5mm or using sensor nominal accuracy	^67,74,75,79,82,91^
	Rating curve uncertainty, calibration and extrapolation	Constraining uncertainty by using a discharge control structure; voting point likelihood; heteroscedastic maximum likelihood model	^56,81,83,85,92^
	Streamflow time discretisation	Comparing indices calculated at different time steps	^5,92^
	Seasonality in hydrological response	Using total length of time series or divided by season	^38,93^
	Hydrological index calculation method, e.g. baseflow separation method	Comparing different algorithms and methods; Correlation analyses; regionalisation	^34,59,64,65,81,87^

**Table 6:** Weir dimensions used to transform water levels to discharge and water level sensor specifications.

**Station**	**90° V-notch section height m**	**Rectangular section width m**	**Sensor range cm H**_**2**_**O**	**Sensor accuracy cm H**_**2**_**O (% FS)**	**Sensor resolution cm H**_**2**_**O (% FS)**
LLO_01_HI_01	0.3030	1.2520	200	±0.12 (±0.06)	0.01 (0.0034)
LLO_02_HI_01	0.3000	1.4000	200	±0.12 (±0.06)	0.01 (0.0034)
JTU_01_HI_01	0.2967	0.8500	200	±0.12 (±0.06)	0.01 (0.0034)
JTU_02_HI_01	0.2961	0.7980	200	±0.12 (±0.06)	0.01 (0.0034)
JTU_03_HI_01	0.2939	0.9000	200	±0.12 (±0.06)	0.01 (0.0034)
JTU_04_HI_01	0.0000	2.9000	200	±0.12 (±0.06)	0.01 (0.0034)
PIU_01_HI_01	0.6000	2.3000	200	±0.12 (±0.06)	0.01 (0.0034)
PIU_02_HI_01	0.6000	2.3000	200	±0.12 (±0.06)	0.01 (0.0034)
PIU_03_HI_01	0.3000	0.9000	200	±0.12 (±0.06)	0.01 (0.0034)
PIU_04_HI_01	0.3000	0.9000	200	±0.12 (±0.06)	0.01 (0.0034)
PIU_07_HI_01	0.3000	0.9000	200	±0.12 (±0.06)	0.01 (0.0034)
CHA_01_HS_01	0.2975	1.2100	1000	±0.5 (±0.05)	Not specified
CHA_02_HS_01	0.2975	1.2000	1000	±0.5 (±0.05)	Not specified
HUA_01_HD_01	1.0000	0.0000	150–1000	±0.50 (±0.05)	0.10–0.20
HUA_01_HD_02	0.3000	1.6000	150–1000	±0.50 (±0.05)	0.10–0.20
HUA_02_HD_01	1.0000	0.0000	150–1000	±0.50 (±0.05)	0.10–0.20
HUA_02_HD_02	0.3000	2.2000	150–1000	±0.50 (±0.05)	0.10–0.20
HMT_01_HI_01	0.2981	0.8950	200	±0.12 (±0.06)	0.01 (0.0034)
HMT_02_HI_01	0.3000	1.5000	200	±0.12 (±0.06)	0.01 (0.0034)
TAM_01_HO_01	0.3065	1.1520	400–900	±0.30–0.50 (± 0.075)	0.14–0.21
TAM_02_HO_01	0.3095	1.1510	400–900	±0.30–0.50 (± 0.075)	0.14–0.21
TIQ_01_HD_01	0.3100	2.1100	150–1000	±0.50–1.00 (±0.10)	0.10–0.20
TIQ_02_HD_01	0.3000	1.2800	150–1000	±0.50–1.00 (±0.10)	0.10–0.20
All V-notch sections used here have a 90-degree angle. FS: full scale.					

## References

[d1] Figshare Ochoa-Tocachi B. F. *et al.* 2018 https://doi.org/10.6084/m9.figshare.c.3943774

